# Recipient Cell Type-Dependent Association of Extracellular Vesicles from Different Cellular Origins

**DOI:** 10.3390/cells15212010

**Published:** 2026-11-04

**Authors:** Doste R. Mamand

**Affiliations:** 1 Department of Laboratory Medicine, Division of Biomolecular and Cellular Medicine, Karolinska Institute, SE-171 77 Stockholm, Sweden; doste.mamand@ki.se; 2 Biology Research Center, Research Center, University of Zakho, Zakho 42002, Duhok, Kurdistan Region, Iraq; 3 Breast Center, Karolinska Comprehensive Cancer Center, Karolinska University Hospital, SE-171 76 Stockholm, Sweden

**Keywords:** EVs, EV-associated fluorescence, recipient-cell identity, nanocarriers, drug delivery, nanomedicine, EV-based therapeutics

## Abstract

Extracellular vesicles (EVs) are promising nanocarriers for therapeutic delivery; however, the factors influencing their interactions with recipient cells remain incompletely understood. This study investigated recipient-cell-specific EV association using mNG-CD81-labelled EVs derived from HEK293T, SKBR-3, and Yummer cells. EV-associated fluorescence was assessed by flow cytometry following incubation with human epithelial, immune, and murine melanoma cell lines at different doses and time points. Marked differences were observed across recipient-cell types. Liver-derived Huh7 and HepG2 cells exhibited the highest percentages of fluorescence-positive cells among human epithelial cells, whereas parental HEK293T cells showed the lowest. THP-1 cells exhibited greater EV-associated fluorescence than Jurkat cells, while Yummer melanoma cells showed higher fluorescence than B16F10 cells following exposure to HEK293T-derived EVs. Similar recipient-cell patterns were observed with SKBR-3- and Yummer-derived EVs. Notably, Yummer cells exhibited relatively high association with their own EVs, demonstrating that low parental-cell association is not universal. EV-associated fluorescence generally increased with dose and incubation time. Although these findings highlight recipient-cell specificity, the relative contributions of EV donor origin and recipient-cell characteristics remain unresolved. Moreover, flow cytometry cannot distinguish surface-associated from internalized EVs. Together, these findings identify recipient-cell specificity as an important determinant of EV-associated fluorescence, providing a foundation for understanding EV–cell interactions and informing the development of targeted EV-based delivery strategies.

## 1. Introduction

Cellular uptake of nanoparticles, including Extracellular Vesicles (EVs), is a fundamental biological process that underpins intercellular communication and the transfer of molecular information between cells [1,2]. EVs are lipid bilayer-enclosed particles naturally released by cells and are known to carry proteins, lipids, and nucleic acids that can modulate the behavior and function of recipient cells [3,4]. Due to these properties, EVs have emerged as promising candidates for applications in diagnostics, drug delivery, and regenerative medicine [5,6]. Historically, extracellular vesicles were initially regarded as cellular debris but are now recognised as important mediators of intercellular communication. The development of EV research has been accompanied by increasing efforts to standardise isolation, characterisation, and reporting practices, particularly through the establishment of the International Society for Extracellular Vesicles (ISEV) and the Minimal Information for Studies of Extracellular Vesicles (MISEV) guidelines. These advances have strengthened the reproducibility and comparability of EV studies [7].

The mechanisms governing EV uptake are complex and multifactorial, involving a range of processes such as clathrin-mediated endocytosis, caveolin-dependent uptake, macropinocytosis, phagocytosis, and direct membrane fusion. These pathways are influenced by both the biochemical composition of EVs and the physiological characteristics of recipient cells [8,9]. Historically, considerable emphasis has been placed on the role of EV origin specifically, the idea that vesicles derived from certain cell types possess inherent targeting capabilities that dictate their uptake specificity [10,11,12]. However, increasing evidence suggests that this perspective may be incomplete. Emerging studies indicate that recipient cell properties such as membrane receptor expression, lipid composition, cytoskeletal organization, and metabolic activity—may play a dominant role in determining EV uptake efficiency. This implies that EV internalization may be less dependent on where the vesicles originate and more dependent on how receptive the target cells are to vesicle interaction and internalization [13,14,15,16]. This shift in understanding has significant implications for both basic biology and translational applications. If EV uptake is primarily governed by recipient cell type, strategies for EV-based therapeutics may need to focus more on modifying or selecting target cells rather than engineering vesicle origin alone [17,18,19,20]. Furthermore, this insight could help explain inconsistencies observed in previous studies where EV uptake did not correlate strongly with vesicle source [21,22,23,24]. Despite these advances, a systematic and controlled comparison of EV uptake across multiple donors and recipient cell combinations remains lacking. Many existing studies are limited by variability in experimental conditions, EV isolation methods, or insufficient cross-comparisons between cell types. Therefore, there is a critical need for a well-controlled investigation that directly addresses whether EV uptake is predominantly dictated by recipient cell identity.

In this study, we conducted a systematic in vitro comparison of EV-associated fluorescence across different recipient-cell types following exposure to EVs derived from HEK293T, SKBR-3, and Yummer cells. By standardising EV preparation, fluorescent labelling, and exposure conditions, we aimed to characterise differences in EV association among human epithelial, immune, and murine melanoma cell lines using flow cytometry, while evaluating the effects of EV dose and incubation time. We also compared patterns of EV association across different donor–recipient combinations. Understanding these interactions may provide insights into recipient-cell specificity and inform future EV-based delivery strategies. We hypothesised that recipient-cell characteristics play a greater role than EV donor-cell origin in determining EV association.

## 2. Materials and Methods

### 2.1. Materials

Dulbecco’s Modified Eagle Medium (DMEM, high glucose; Catalog No. 11965092), Roswell Park Memorial Institute medium (RPMI-1640; Catalog No. 11875093), fetal bovine serum (FBS), serum-free Opti-MEM (Catalog No. 31985070), and phosphate-buffered saline (PBS; Catalog No. 10010015) were obtained from Gibco (New York 14072, USA). A 1% antibiotic–antimycotic (anti-anti) solution (Catalog No. 15240062) was purchased from Gibco (New York 14072,USA). Mycoplasma detection kits were sourced from Eurofins and routinely used to screen cell cultures for contamination.

### 2.2. Cell Culture

Human embryonic kidney cells (HEK293T), SKBR-3 human breast cancer cells, pancreatic ductal adenocarcinoma (PDAK) cells, HepG2 human hepatic cancer cells, Huh7 human hepatocellular carcinoma cells, Caco-2 human colorectal adenocarcinoma cells, and the murine melanoma cell lines B16F10 and Yummer (all obtained from ATCC) were cultured in DMEM supplemented with 10% FBS and 1% antibiotic–antimycotic solution. Cells were maintained in 150 mm tissue culture dishes (standard surface; Sarstedt, Cat. No. 83.3903.300) at 37 °C in a humidified incubator with 5% CO_2_ for 24 h to permit cell attachment and recovery. Subsequently, the culture medium was replaced with serum-free Opti-MEM containing 1% antibiotic–antimycotic solution to enable EV production under serum-free conditions. The conditioned medium (CM) was harvested after 48 h, following which it was purified and concentrated for subsequent use.

### 2.3. Plasmid Generation

Plasmids encoding human CD81 or mouse CD81 fused to mNeonGreen (mNG) were generated using the lentiviral p2CL9IPw5 backbone vector. The amino acid sequences for human CD81, mouse CD81, and mNG were verified using UniProt. Synthesized gene fragments (Integrated DNA Technologies) were cloned downstream of the spleen focus-forming virus (SFFV) promoter within the p2CL9IPw5 vector using standard restriction enzyme cloning strategies [25]. The constructs additionally contained an internal ribosomal entry site (IRES)-puromycin resistance cassette for stable cell selection. All plasmid constructs were verified by Sanger sequencing performed by Eurofins Genomics prior to use in lentiviral production.

### 2.4. Generating HEK293T, SKBR-3, and Yummer Cells Stably Expressing CD81-mNG EVs

Lentiviral supernatants were generated as previously described in reference [25]. Briefly, HEK293T cells were co-transfected with the p2CL9IPw5 construct encoding human or mouse CD81 tagged with mNeonGreen (mNG), the pCD/NL-BH helper construct, and the pcoPE human foamy virus envelope construct using polyethyleneimine (PEI). At 18 h post-transfection, expression from the human cytomegalovirus immediate-early enhancer/promoter was enhanced by treating the cells with 10 mM sodium butyrate (Sigma-Aldrich) for 7 h. The medium was then replaced, and the viral supernatant was collected 22 h later. Viral particles were concentrated by centrifugation at 25,000× *g* for 90 min at 4 °C. After removing the supernatant, the viral pellet was resuspended in 1 mL Iscove’s Modified Dulbecco’s Medium supplemented with 20% FBS and 1% antibiotic–antimycotic solution. Virus stocks were stored at −80 °C until use. Cells were then transduced with the lentivirus and cultured under puromycin selection for at least five passages to generate stable cell lines.

### 2.5. Isolation of EVs

HEK293T, SKBR-3, and Yummer cells were seeded at a density of 1 × 10^7^ cells in 20 mL DMEM per 150 × 20 mm Petri dish (SARSTEDT AG & Co. KG, Nümbrecht, Germany) and cultured under the conditions described previously [25]. After 24 h, the culture medium was replaced with serum-free Opti-MEM. Following an additional 48 h incubation period, the conditioned media (CM) were collected for subsequent EV isolation and purification as described below.

### 2.6. Purification of EVs

EVs were isolated from conditioned media (CM) using sequential centrifugation, filtration, ultrafiltration, and size exclusion chromatography (SEC). Initially, the CM was pre-cleared by centrifugation at 700× *g* for 5 min, followed by centrifugation at 2000× *g* for 10 min to remove cells, debris, and larger particles. Unless otherwise specified, samples were subsequently filtered through a 0.22 µm cellulose acetate bottle-top filter (SARSTEDT AG & Co. KG, Germany) to eliminate remaining large particles.

The filtered CM was concentrated by tangential flow filtration using a MicroKross ultrafiltration module (20 cm^2^, 300 kDa molecular weight cut-off; Spectrum Laboratories, Rancho Dominguez, CA, USA). The resulting retentate was further concentrated using Amicon Ultra-15 centrifugal filter units with a 10 kDa molecular weight cut-off (MilliporeSigma, Burlington, MA, USA) to a final volume of 100 µL, as previously described by Mamand et al. [26]. Subsequently, EVs were purified by size exclusion chromatography using qEV 70 nm columns for EV isolation (Izon Science, Christchurch, New Zealand). Fractions 4 and 5 were collected in a total volume of 2 mL and further concentrated using Amicon^®^ Ultra-15 centrifugal filter units (10 kDa; Merck, Darmstadt, Germany). Purified EV preparations were stored at −80 °C until further analysis.

### 2.7. Nanoparticle Tracking Analysis

Nanoparticle tracking analysis (NTA) measurements were performed using the Particle Metrix ZetaView system as performed before [25,27]. Samples were diluted in 0.22 µm-filtered PBS to a final volume of 1 mL. Optimal measurement concentrations were established through preliminary testing to obtain particle counts ranging from 140 to 200 particles per frame. The manufacturer’s recommended default software settings for EV analysis were applied. These settings included a camera sensitivity of 80 and a shutter speed of 100. For each sample, two measurement cycles were carried out across 11 cell positions, with 60 frames recorded per position using the high video setting. Measurement parameters were configured as follows: autofocus enabled, camera sensitivity set to 80.0, shutter speed maintained at 100, scattering intensity adjusted to 4.0, and cell temperature maintained at 24 °C. Following video acquisition, the recorded data were processed and analyzed using the integrated ZetaView Software version 8.02.31.

### 2.8. Electron Microscopy of EV Morphology

Three microliters of sample were applied to glow-discharged, carbon-coated, Formvar-stabilized 400-mesh copper grids (easiGlow™, Ted Pella, Redding, CA, USA) and incubated for approximately 30 s [25]. Excess sample was blotted off, followed by a Milli-Q water wash. The grids were negatively stained with 1% ammonium molybdate and imaged using an HT7800-Xarosa transmission electron microscope (TEM; Hitachi High-Technologies, Tokyo, Japan) operated at 80 kV and equipped with a 4 MP Veleta CCD camera (Olympus Soft Imaging Solutions GmbH, Münster, Germany).

### 2.9. Imaging Flow Cytometry for Characterization of EV Surface Markers

EV surface marker characterization was performed using imaging flow cytometry as previously described [26]. Purified EVs were diluted in Dulbecco’s phosphate-buffered saline (DPBS) to a concentration of 1 × 10^10^ particles/mL. A total of 2.5 × 10^8^ EVs in 25 µL were incubated with 8 nM allophycocyanin (APC)-conjugated antibodies against the EV-associated surface marker CD81 (Miltenyi Biotec, Bergisch Gladbach, Germany). Samples were incubated overnight at room temperature in the dark in sealed 96-well plates (SARSTEDT AG & Co. KG, Germany). Following incubation, the samples were diluted in 1% human albumin and trehalose buffer (PBS-HAT), as previously described [27], to achieve a final concentration of 1 × 10^7^ EVs/mL in a total volume of 100 µL without further antibody addition. Single-vesicle imaging and small-particle analysis were carried out using the Amnis CellStream imaging flow cytometer from Luminex Corporation. Data acquisition and analysis were performed using FlowJo v10.8 software (FlowJo LLC, Ashland, OR, USA).

### 2.10. Western Blotting for EV Markers

Western blotting was performed to evaluate the presence of the intravesicular protein TSG101 in EVs derived from HEK293T and SKBR-3 cells [19]. Briefly, 2 × 10^10^ EVs (equivalent to approximately 15 µg EV protein) and 2 × 10^6^ HEK293T or SKBR-3 cells were lysed in 100 µL radioimmunoprecipitation assay (RIPA) buffer (Bio-Rad Laboratories, Hercules, CA, USA). Lysates were incubated on ice for 30 min with intermittent vortexing for 10 s every 5 min. The EV and cell lysates were mixed with 8 µL loading buffer composed of 10% glycerol, 8% sodium dodecyl sulfate (SDS), 0.5 M dithiothreitol (DTT), and 0.4 M sodium carbonate. Samples were heated at 70 °C for 5 min prior to separation on NuPAGE™ 4–12% Bis-Tris gels (Invitrogen) and electrophoresed at 120 V for 1.5 h. Proteins were subsequently transferred to iBlot transfer membranes using the iBlot 2 Dry Blotting System for 7 min. Membranes were blocked for 1 h at room temperature using Odyssey Blocking Buffer (LI-COR Biosciences, Lincoln, NE, USA) and incubated overnight at 4 °C with freshly prepared anti-TSG101 primary antibody (MA1-23296, Invitrogen) diluted 1:500. Following four washes with 0.1% TBS-T for 5 min each under agitation, membranes were incubated for 1 h with a 1:10,000 diluted secondary antibody (goat anti-human, H10007). After three PBS washes, protein bands were visualized using the LI-COR Odyssey CLx Imaging System.

### 2.11. Assessment of EV-Associated Fluorescence by Flow Cytometry

For each uptake experiment, 15,000 cells from each cell line were seeded per well in 96-well plates (Catalog No. 83.3924.005, Sarstedt). Adherent cells were cultured in complete DMEM, whereas suspension cells were maintained in complete RPMI medium. After 24 h of incubation, EVs were added at concentrations of 1 × 10^8^, 1 × 10^9^, 5 × 10^9^, and 1 × 10^10^ EVs per well. Cells were incubated with EVs for 2 h or 4 h at 37 °C under standard cell culture conditions. Untreated control cells were maintained under the same experimental conditions as EV-treated cells but without exposure to mNG-labeled EVs. Following incubation, cells were washed twice with PBS to remove unbound EVs. Adherent cells were detached by trypsinization for 5 min, while suspension cells were collected directly after washing. Cells were subsequently resuspended in fresh culture medium and analyzed by flow cytometry using a MACSQuant 16 flow cytometer (Miltenyi Biotec). At least 5000 to 10,000 events were recorded per sample. Forward and side scatter parameters were used to gate viable cell populations and exclude debris, and fluorescence intensity was measured to evaluate EV-associated fluorescence by recipient cells. Data acquisition and analysis were performed using FlowJo v10.8 software (FlowJo LLC).

### 2.12. Statistical Analysis

Data are presented as mean ± standard deviation (SD) from three independent biological replicates (n = 3). Each biological replicate consisted of an independently produced and isolated EV preparation obtained at a different time point. One technical replicate well was used for each EV preparation, and their measurements were averaged to obtain a single value per biological replicate. No data were excluded from the analyses. Statistical analyses were performed using two-way analysis of variance (ANOVA), followed by Tukey’s multiple-comparisons test to compare all group means within each row, with adjustment for multiple comparisons. Statistical significance was defined as an adjusted *p*-value < 0.05. All analyses were conducted using GraphPad Prism version 10.

## 3. Results

**Figure 1:**
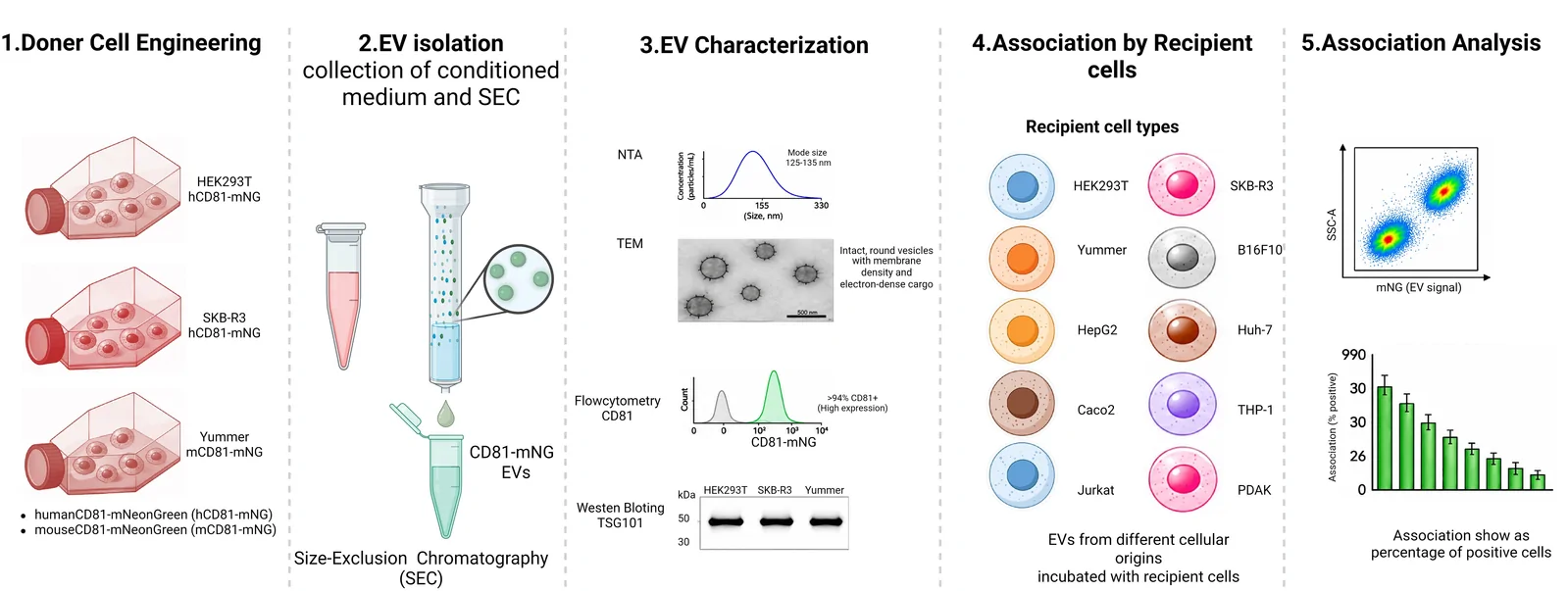
Experimental workflow for EV generation, isolation, characterization, and cell association analysis. Schematic overview of the experimental design used to investigate EV association across multiple recipient cell types. Donor cell lines (HEK293T, SKBR-3, and Yummer) were genetically engineered to express CD81 fused to mNeonGreen (mNG), enabling fluorescent labeling of secreted EVs. Engineered donor cells were cultured under EV-production conditions, and conditioned media were collected for EV isolation. EVs were purified using size exclusion chromatography (SEC) prior to characterization. Characterization included nanoparticle tracking analysis (NTA) for size distribution, transmission electron microscopy (TEM) for vesicle morphology, imaging flow cytometry for fluorescence and CD81 expression, and Western blot analysis for EV-associated markers. Purified fluorescent EVs were subsequently incubated with different recipient cell lines, including epithelial, immune, and melanoma-derived cells. EV association was quantified by flow cytometry based on mNG fluorescence intensity within recipient cells. The figure was created by Biorender.

To investigate the uptake of EVs between cell lines in vitro, three donor cell lines were transduced with a lentivirus encoding CD81-mNeonGreen (mNG). This generated stable cell lines expressing fluorescently labeled CD81-mNG EVs (Figure 1, Step 1). The engineered donor cells were subsequently cultured in EV-free serum media to enable secretion of mNG-labeled EVs into the conditioned medium. The released EVs were then isolated and purified by SEC as illustrated in Figure 1 (Step 2). Following isolation, the purified EVs were characterized to confirm their quality, size distribution, and marker expression, as shown in Figure 1 (Step 3). Next, ten different recipient cancer cell lines were utilized for the uptake study, including HEK293T, SKBR-3, Yummer, B16F10, HepG2, Huh7, Caco-2, THP-1, Jurkat, and PDAK, as indicated in Figure 1 (Step 4). In the final step, EV uptake by recipient cells was evaluated using fluorescence-based analysis to determine differences in cellular internalization efficiency among the tested cell lines.

### 3.1. Characterisation of EVs

**Figure 2:**
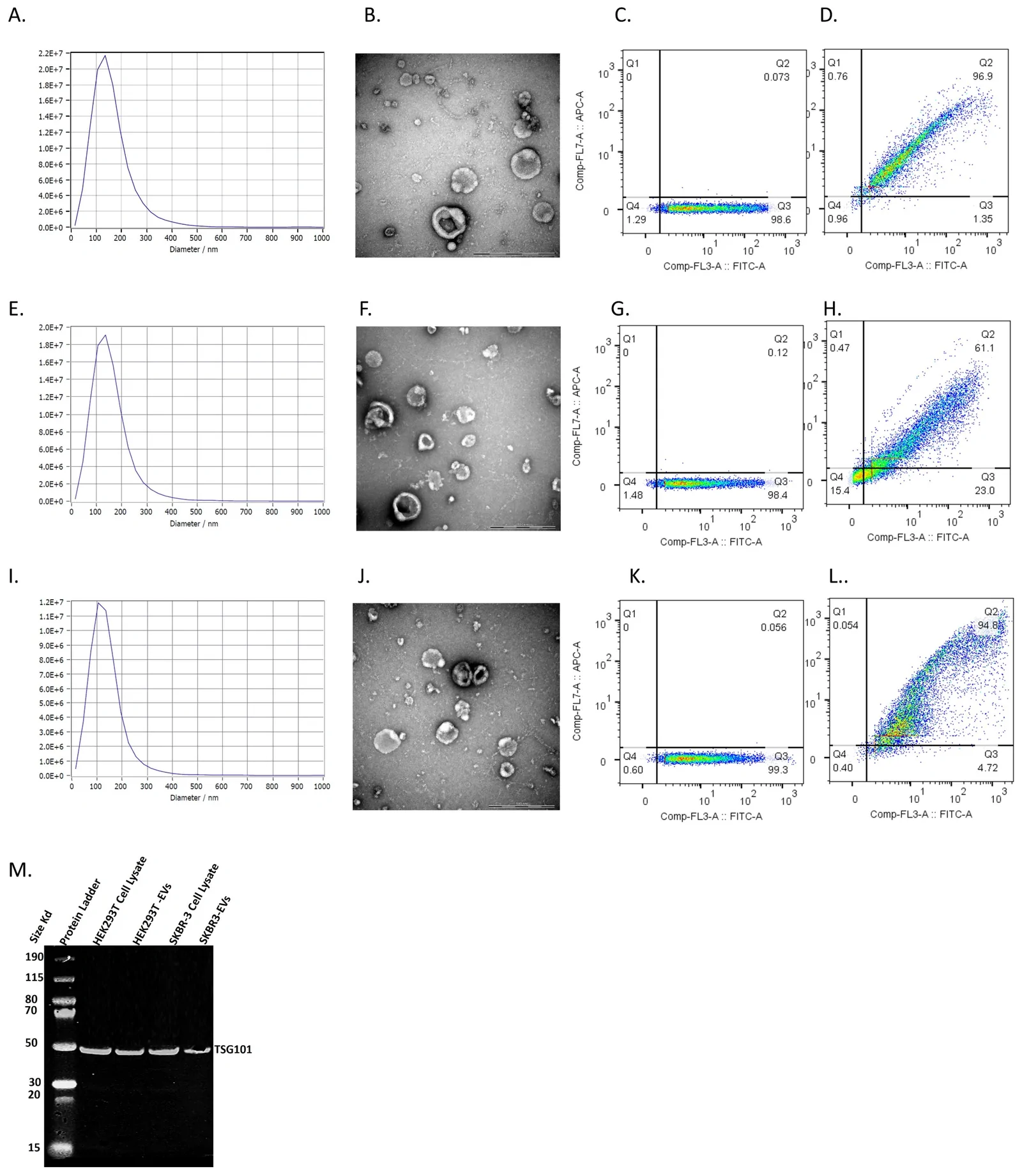
Characterization of extracellular vesicles isolated from engineered donor cell lines. (**A**) Nanoparticle tracking analysis (NTA) showing the size distribution profile of HEK293T-derived mNG-labeled EVs. (**B**) Transmission electron microscopy (TEM) image of HEK293T-derived EVs showing intact membrane-bound vesicles. Scale bar = 500 nm. (**C**) Imaging flow cytometry analysis of HEK293T-derived EVs demonstrating fluorescent mNG-positive vesicles. (**D**) Imaging flow cytometry analysis of HEK293T-derived EVs stained with APC-conjugated anti-human CD81 antibodies. (**E**) NTA size distribution profile of SKBR-3-derived EVs. (**F**) TEM image of SKBR-3-derived EVs. Scale bar = 500 nm. (**G**) Imaging flow cytometry analysis of SKBR-3-derived EVs. (**H**) Imaging flow cytometry analysis of SKBR-3-derived EVs stained with APC-conjugated anti-human CD81 antibodies. (**I**) NTA size distribution profile of Yummer-derived EVs. (**J**) TEM image of Yummer-derived EVs. Scale bar = 500 nm. (**K**) Imaging flow cytometry analysis of Yummer-derived EVs. (**L**) Imaging flow cytometry analysis of Yummer-derived EVs stained with APC-conjugated anti-mouse CD81 antibodies. (**M**) Western blot analysis of TSG101 in HEK293T-derived and SKBR-3-derived EV preparations.

To further investigate the potential role of EVs in cellular uptake across different cell lines, EVs were isolated from cultured engineered HEK293T, SKBR-3, and Yummer cells expressing mNG-hCD81 or mNG-mCD81. Vesicles were purified using SEC and subsequently characterized using complementary analytical techniques. Nanoparticle tracking analysis (NTA) demonstrated that the isolated EVs had a modal particle size ranging from 125 to 135 nm, consistent with the expected size distribution of small EVs (Figure 2A,E,I).

Morphological examination by transmission electron microscopy (TEM) confirmed the presence of both small and larger vesicle populations that were predominantly intact, spherical, and membrane bound. Distinct membrane density at the lipid bilayer, together with electron-dense intravesicular cargo, was also visible, further supporting successful EV isolation (Figure 2B,F,J). The size heterogeneity observed by TEM is consistent with the EV size distributions obtained by NTA. Minor differences may arise from the distinct measurement principles, as NTA provides a population-level size distribution of particles in suspension, whereas TEM enables direct visualization of individual vesicles. Additionally, sample dehydration during TEM preparation may influence the apparent vesicle size. Flow cytometric analysis revealed that over 94% of the detected vesicles were positive for the canonical EV surface marker CD81, indicating a highly enriched EV population with strong marker expression (Figure 2C,D,G,H,K,L). In addition, Western blot analysis confirmed the presence of tumour susceptibility gene 101 (TSG101), a well-established EV-associated protein, in EV preparations derived from HEK293T and SKBR-3 cells (Figure 2M). Collectively, support the successful isolation of EVs using SEC, with particle characteristics and EV-associated marker expression consistent with extracellular vesicles, supporting their use in subsequent EV association studies.

### 3.2. Assessment of HEK293T-Derived EV Association with Human Epithelial Cell Lines

**Figure 3:**
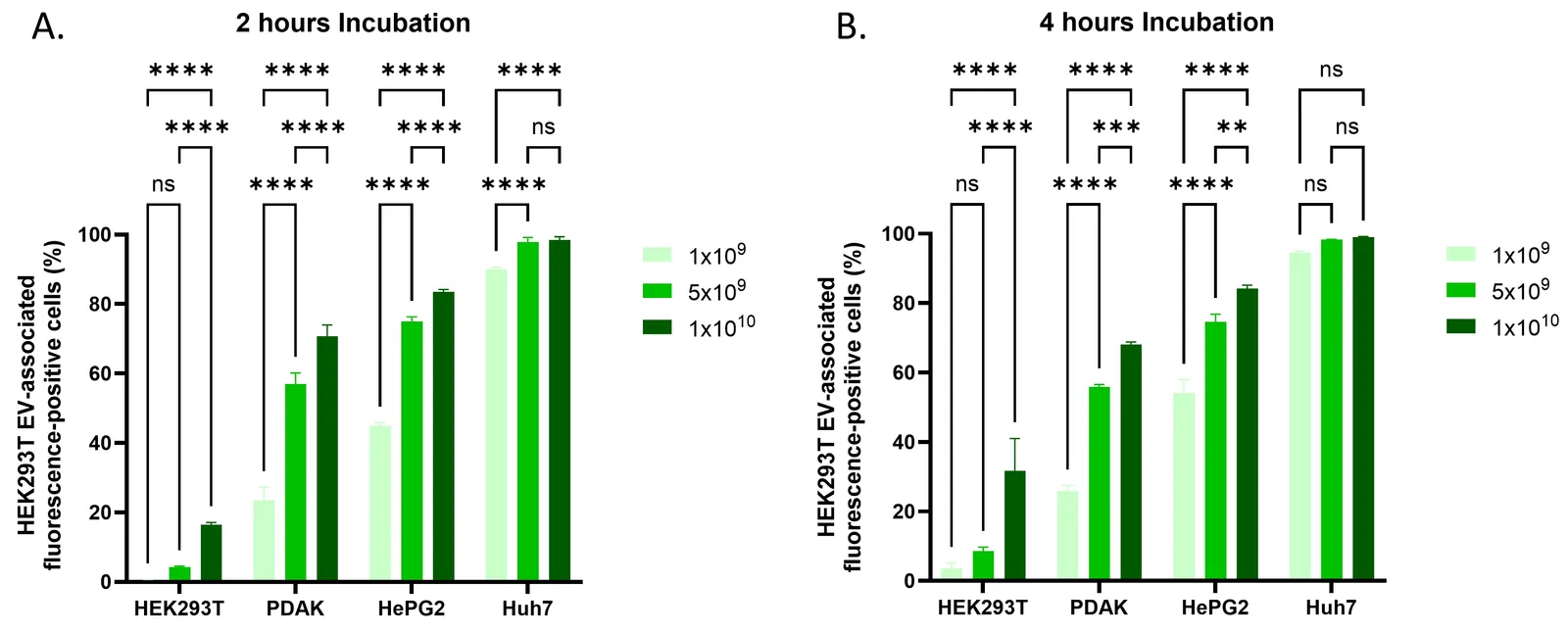
Cell-associated fluorescence following exposure to HEK293T-derived EVs in human epithelial cell lines. (**A**) Percentage of cells exhibiting EV-associated fluorescence in HEK293T, PDAK, HepG2, and Huh7 cells following 2 h of incubation with HEK293T-derived mNG-CD81-labelled EVs at concentrations of 1 × 10^9^, 5 × 10^9^, and 1 × 10^10^ particles. (**B**) Percentage of cells exhibiting EV-associated fluorescence following 4 h of incubation at the indicated concentrations. Cell-associated fluorescence was measured by flow cytometry following incubation and washing and does not necessarily indicate EV internalization. Data are presented as mean ± standard deviation (SD) from three independent biological replicates (n = 3), each representing an independently produced and isolated EV preparation obtained at a different time point. Statistical analyses were performed using two-way analysis of variance (ANOVA), followed by Tukey’s multiple-comparisons test to compare group means within each row, with adjustment for multiple comparisons. Statistical significance is indicated as follows: ns, not significant; ** *p* < 0.01; *** *p* < 0.001; **** *p* < 0.0001.

Given that HEK293T-derived EVs are widely used in preclinical applications, it is important to understand how their association with recipient cells varies across different cell types. To investigate this, cell-associated fluorescence following exposure to HEK293T-derived mNG-CD81-labelled EVs was evaluated in several human epithelial cell lines in vitro. The results demonstrated that the percentage of cells exhibiting EV-associated fluorescence varied substantially among cell lines and was influenced by both incubation time and EV dose. Experiments were performed at two time points (2 h and 4 h) using three EV concentrations (1 × 10^9^, 5 × 10^9^, and 1 × 10^10^ particles). At the 2 h time point, liver-derived cell lines exhibited the highest percentages of cells with EV-associated fluorescence. Huh7 cells showed the highest percentages, with values of 90%, 98%, and 98.5% following treatment with 1 × 10^9^, 5 × 10^9^, and 1 × 10^10^ EVs, respectively. HepG2 cells showed the second-highest percentages, reaching 45%, 75%, and 85% at the respective doses. PDAK cells exhibited values of 23%, 57%, and 71%. The lowest percentages of fluorescence-positive cells were observed in HEK293T cells, the parental cell line from which the EVs were derived, with values of 0.6%, 4.5%, and 16.5% at the three tested doses (Figure 3A, Supplementary Figure S1). At the 4 h time point, the percentage of cells exhibiting EV-associated fluorescence generally increased. In Huh7 cells, the percentages approached 100%, reaching 95%, 98.3%, and 99% at the three respective doses. HEK293T cells also showed an increase over time, with values of 3.5%, 8.5%, and 31.7%. In contrast, PDAK cells exhibited no notable change between the two time points. Similarly, HepG2 cells-maintained values of 75% and 85% at the 5 × 10^9^ and 1 × 10^10^ EV doses, respectively, whereas the percentage of fluorescence-positive cells at 1 × 10^9^ EVs increased by approximately 10% (Figure 3B, Supplementary Figure S1). Overall, these findings indicate that the percentage of cells exhibiting HEK293T-derived EV-associated fluorescence varied considerably among recipient cell lines, with liver-derived cell lines showing the highest percentages and parental HEK293T cells showing the lowest under the experimental conditions examined.

### 3.3. Assessment of HEK293T-Derived EV Association with Human Immune Cell Lines

**Figure 4:**
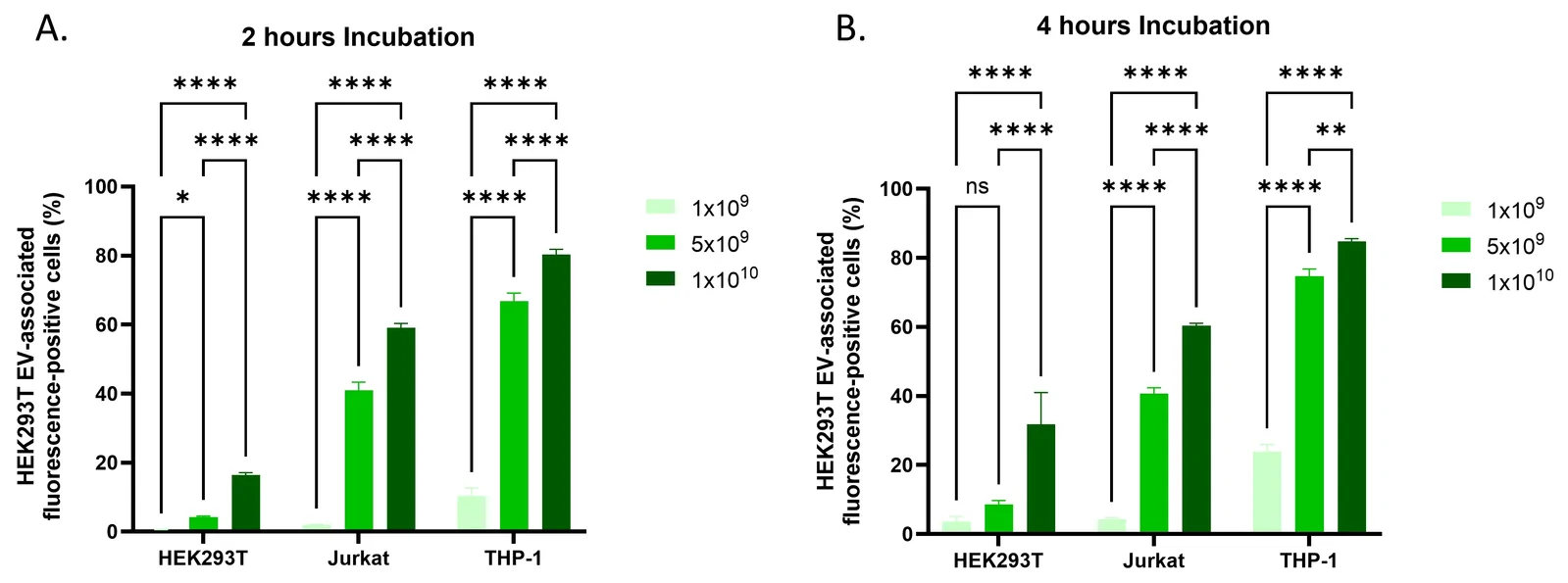
Cell-associated fluorescence following exposure to HEK293T-derived EVs in human immune and reference cell lines. (**A**) Percentage of cells exhibiting EV-associated fluorescence in Jurkat, THP-1, and HEK293T cells following 2 h of incubation with HEK293T-derived mNG-CD81-labelled EVs at concentrations of 1 × 10^9^, 5 × 10^9^, and 1 × 10^10^ particles. (**B**) Percentage of cells exhibiting EV-associated fluorescence following 4 h of incubation at the indicated concentrations. Cell-associated fluorescence was measured by flow cytometry following incubation and washing and does not necessarily indicate EV internalization. Data are presented as mean ± standard deviation (SD) from three independent biological replicates (n = 3), each representing an independently produced and isolated EV preparation obtained at a different time point. Statistical analyses were performed using two-way analysis of variance (ANOVA), followed by Tukey’s multiple-comparisons test to compare group means within each row, with adjustment for multiple comparisons. Statistical significance is indicated as follows: ns, not significant; * *p* < 0.05; ** *p* < 0.01; **** *p* < 0.0001.

Next, the association of HEK293T-derived mNG-CD81-labelled EVs with human immune cell lines was evaluated by flow cytometry. THP-1 and Jurkat cells were evaluated in parallel with HEK293T cells, which served as a reference control to assess EV association with their parental cells. Overall, both immune cell lines exhibited higher percentages of cells with EV-associated fluorescence than HEK293T cells. At the 2 h time point, THP-1 cells exhibited the highest percentage of fluorescence-positive cells among the tested cell lines, with values of 10.33%, 66.8%, and 80.3% following treatment with 1 × 10^9^, 5 × 10^9^, and 1 × 10^10^ EVs, respectively. Jurkat cells also showed substantial EV-associated fluorescence, with 2%, 41%, and 59% of cells exhibiting detectable fluorescence at the respective doses. In contrast, HEK293T cells displayed the lowest percentages of fluorescence-positive cells, as illustrated in Figure 4A and Supplementary Figure S2.

At the 4 h time point, THP-1 cells showed a further increase in the percentage of fluorescence-positive cells, reaching 23.8%, 74.7%, and 84.7% at the three respective doses. HEK293T cells also demonstrated an increase over time, with values of 3.5%, 8.5%, and 31.7% following treatment with 1 × 10^9^, 5 × 10^9^, and 1 × 10^10^ EVs, respectively. However, Jurkat cells showed minimal changes compared with the 2 h time point, with only a slight increase (approximately 2%) observed at the lowest EV dose (1 × 10^9^), while the percentages of fluorescence-positive cells at higher doses remained largely unchanged, as illustrated in Figure 4B and Supplementary Figure S2. Overall, these results indicate that a higher proportion of THP-1 and Jurkat cells exhibited EV-associated fluorescence compared with parental HEK293T cells, with THP-1 cells consistently showing the highest percentages under the conditions examined.

### 3.4. Assessment of HEK293T-Derived EV Association with Murine Melanoma Cell Lines

**Figure 5:**
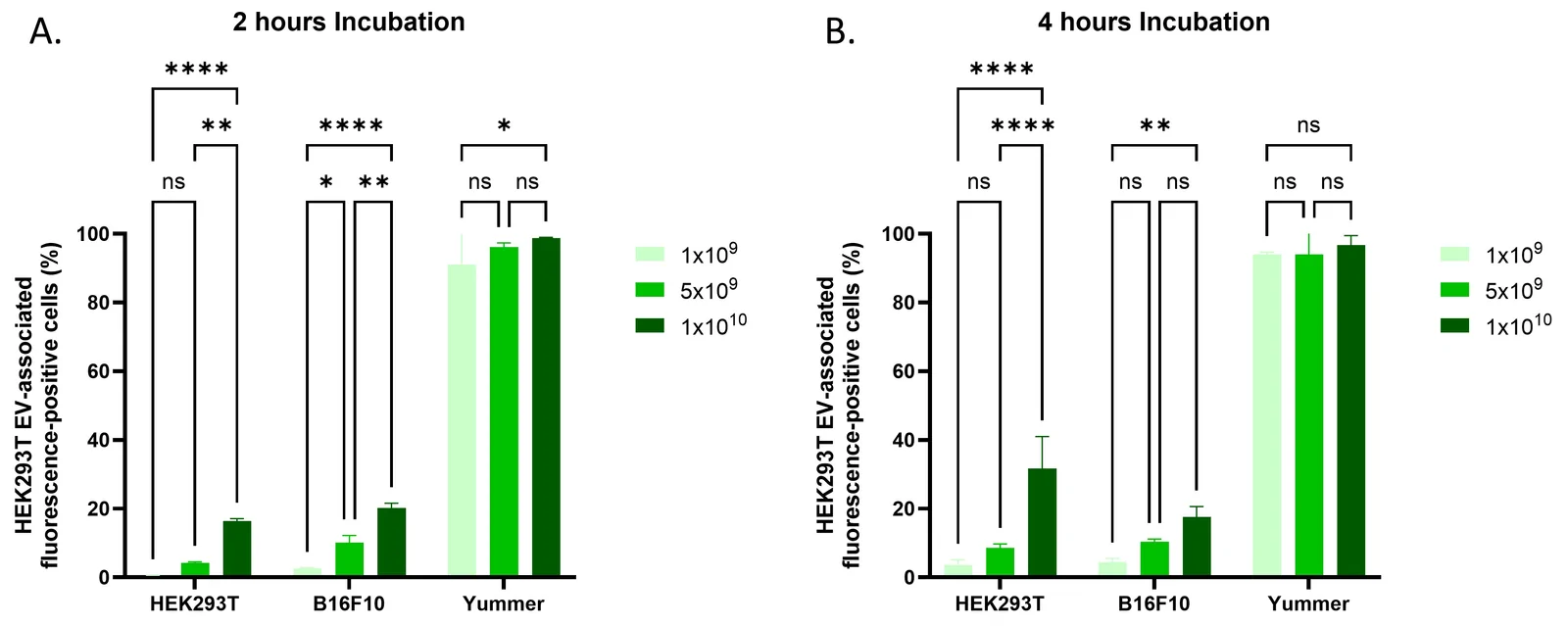
Cell-associated fluorescence following exposure to HEK293T-derived EVs in murine melanoma cell lines. (**A**) Percentage of cells exhibiting EV-associated fluorescence in B16F10 and Yummer melanoma cells following 2 h of incubation with HEK293T-derived mNG-CD81-labelled EVs at concentrations of 1 × 10^9^, 5 × 10^9^, and 1 × 10^10^ particles. (**B**) Percentage of cells exhibiting EV-associated fluorescence following 4 h of incubation at the indicated concentrations. Cell-associated fluorescence was measured by flow cytometry following incubation and washing and does not necessarily indicate EV internalization. Data are presented as mean ± standard deviation (SD) from three independent biological replicates (n = 3), each representing an independently produced and isolated EV preparation obtained at a different time point. Statistical analyses were performed using two-way analysis of variance (ANOVA), followed by Tukey’s multiple-comparisons test to compare group means within each row, with adjustment for multiple comparisons. Statistical significance is indicated as follows: ns, not significant; * *p* < 0.05; ** *p* < 0.01; **** *p* < 0.0001.

As described above, the percentage of cells exhibiting HEK293T-derived EV-associated fluorescence varied among human cell lines. To determine whether similar differences could be observed in murine cells, two mouse melanoma cell lines, B16F10 and Yummer, were evaluated in vitro in parallel with HEK293T cells. The results showed clear differences in the percentages of cells exhibiting EV-associated fluorescence between the tested cell lines. Yummer cells exhibited higher percentages of fluorescence-positive cells following exposure to HEK293T-derived mNG-CD81-labelled EVs than both B16F10 and HEK293T cells. In contrast, B16F10 cells showed lower percentages of fluorescence-positive cells than Yummer cells. These findings suggest that recipient cell type is associated with differences in EV-associated fluorescence. This trend was consistently observed at both examined time points (2 h and 4 h) (Figure 5, Supplementary Figure S3). Furthermore, the percentage of fluorescence-positive cells generally increased with increasing EV concentration. However, no significant differences were observed between the higher EV doses under some experimental conditions (Figure 5, Supplementary Figure S3).

### 3.5. Assessment of SKBR-3-Derived EV Association with Human Epithelial Cell Lines

**Figure 6:**
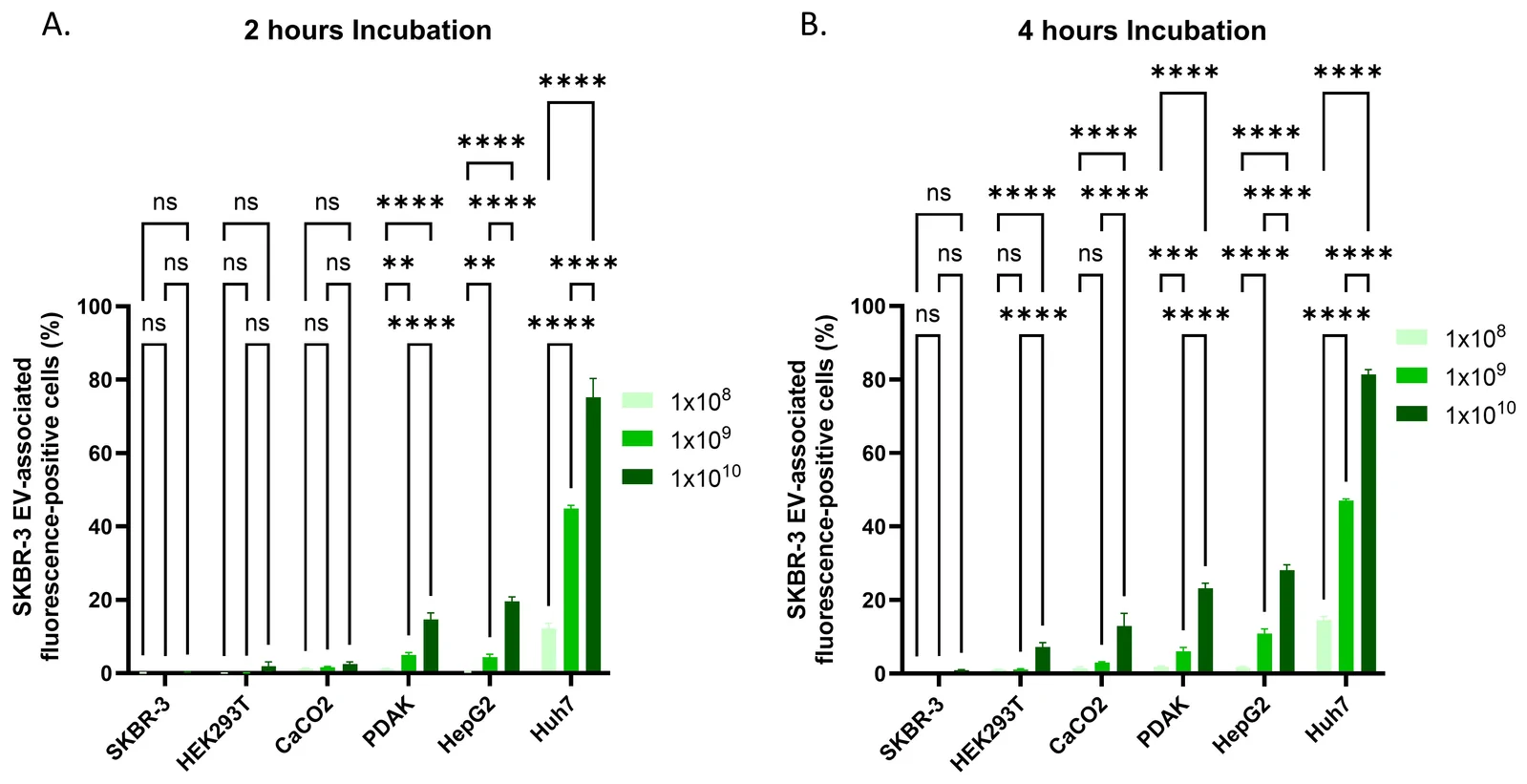
Cell-associated fluorescence following exposure to SKBR-3-derived EVs in human epithelial cell lines. (**A**) Percentage of cells exhibiting EV-associated fluorescence in SKBR-3, HEK293T, Caco-2, PDAK, HepG2, and Huh7 cells following 2 h of incubation with SKBR-3-derived mNG-CD81-labelled EVs at concentrations of 1 × 10^8^, 1 × 10^9^, and 1 × 10^10^ particles. (**B**) Percentage of cells exhibiting EV-associated fluorescence following 4 h of incubation at the indicated concentrations. Cell-associated fluorescence was measured by flow cytometry following incubation and washing and does not necessarily indicate EV internalization. Data are presented as mean ± standard deviation (SD) from three independent biological replicates (n = 3), each representing an independently produced and isolated EV preparation obtained at a different time point. Statistical analyses were performed using two-way analysis of variance (ANOVA), followed by Tukey’s multiple-comparisons test to compare group means within each row, with adjustment for multiple comparisons. Statistical significance is indicated as follows: ns, not significant; ** *p* < 0.01; *** *p* < 0.001;**** *p* < 0.0001.

To investigate whether EV donor-cell origin influences EV association with recipient cells, SKBR-3 cells were transduced with a lentiviral construct encoding mNG-CD81 to generate fluorescently labelled EVs, as previously described for HEK293T cells. SKBR-3-derived mNG-CD81-labelled EVs were then incubated with a panel of human epithelial cell lines, including Caco-2, PDAK, HEK293T, SKBR-3, HepG2, and Huh7 cells. The results demonstrated that Huh7 and HepG2 cells exhibited the highest percentages of cells with EV-associated fluorescence following exposure to SKBR-3-derived EVs, exceeding those observed in the EV-producing SKBR-3 cells. PDAK cells also showed increases in the percentage of fluorescence-positive cells with increasing EV dose and incubation time. A similar pattern was observed in Caco-2 cells, with differences evident across EV doses and incubation times. HEK293T cells displayed lower percentages of fluorescence-positive cells than most of the tested cell lines. However, after 4 h of incubation, the percentage of fluorescence-positive HEK293T cells was significantly higher than that of SKBR-3 cells. Overall, these findings demonstrate that EV-associated fluorescence varied among recipient cell lines following exposure to SKBR-3-derived EVs. The relatively high percentages observed in Huh7 and HepG2 cells were consistent with the patterns observed following exposure to HEK293T-derived EVs (Figure 6, Supplementary Figure S4).

### 3.6. Assessment of Yummer-Derived EV Association with Murine Melanoma Cell Lines

**Figure 7:**
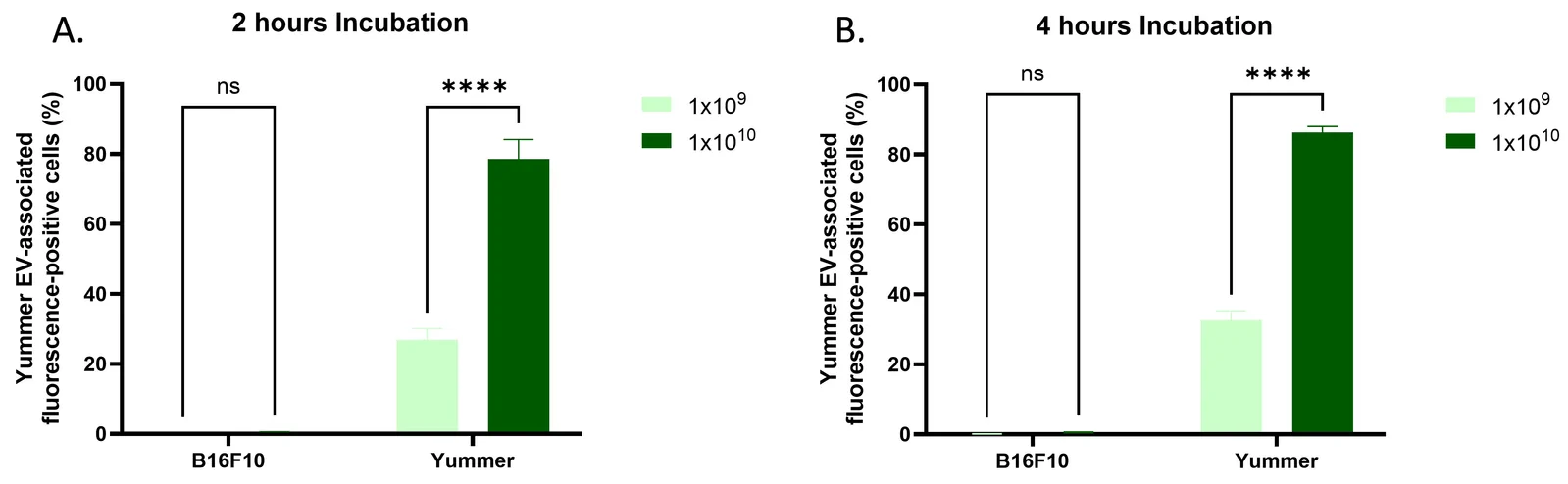
Cell-associated fluorescence following exposure to Yummer-derived EVs in murine melanoma cell lines. (**A**) Percentage of cells exhibiting EV-associated fluorescence in B16F10 and Yummer cells following 2 h of incubation with Yummer-derived mNG-CD81-labelled EVs at concentrations of 1 × 10^9^ and 1 × 10^10^ particles. (**B**) Percentage of cells exhibiting EV-associated fluorescence following 4 h of incubation at the indicated concentrations. Cell-associated fluorescence was measured by flow cytometry following incubation and washing and does not necessarily indicate EV internalization. Data are presented as mean ± standard deviation (SD) from three independent biological replicates (n = 3), each representing an independently produced and isolated EV preparation obtained at a different time point. Statistical analyses were performed using two-way analysis of variance (ANOVA), followed by Tukey’s multiple-comparisons test to compare group means within each row, with adjustment for multiple comparisons. Statistical significance is indicated as follows: ns, not significant; **** *p* < 0.000.

Based on the relatively low EV-associated fluorescence observed in HEK293T and SKBR-3 parental cells following exposure to their respective EVs, we hypothesised that EVs might exhibit limited association with their cells of origin. To investigate this possibility, Yummer cells were transduced with a lentiviral construct encoding mNG-CD81 to generate fluorescently labelled EVs, which were subsequently incubated with Yummer and B16F10 melanoma cells. Interestingly, Yummer cells exhibited significantly higher EV-associated fluorescence than B16F10 cells (Figure 7 and Supplementary Figure S5), indicating that low parental-cell EV association is not a universal phenomenon. Yummer-derived mNG-CD81-labelled EVs were also incubated with human recipient-cell lines, including HEK293T, SKBR-3, Jurkat, PDAK, HepG2, and Huh7. The patterns of EV-associated fluorescence across these cell lines were broadly similar to those observed with HEK293T- and SKBR-3-derived EVs (Supplementary Figure S6). Collectively, these findings suggest that recipient-cell identity is associated with differences in EV-associated fluorescence across the donor–recipient combinations examined. However, the relative contributions of donor-cell origin and recipient-cell characteristics remain unresolved and require further investigation.

## 4. Discussion

EVs are increasingly used as delivery platforms in preclinical and translational research because of their ability to transport bioactive cargo to recipient cells [28]. However, efficient therapeutic application requires a clear understanding of how EVs are internalized by different target cells [29]. In the present study, we compared cell-associated fluorescence following exposure to HEK293T-, SKBR-3-, and Yummer-derived mNG-CD81-labelled EVs across human epithelial and immune cell lines and murine melanoma models. Our findings revealed substantial differences in EV-associated fluorescence among recipient-cell types, with dose- and time-dependent changes observed under several experimental conditions. Importantly, because fluorescence was assessed by flow cytometry following incubation and washing, these measurements reflect cell-associated EV-derived fluorescence rather than confirmed EV internalization.

One of the most striking findings was the high percentage of fluorescence-positive cells observed in liver-derived epithelial cell lines, particularly Huh7 and HepG2 cells. Following exposure to HEK293T-derived EVs, Huh7 cells exhibited 90–98.5% fluorescence-positive cells after 2 h, increasing to 95–99% after 4 h across the tested doses. HepG2 cells also exhibited relatively high percentages compared with several other recipient-cell lines. Similar patterns were observed following exposure to SKBR-3-derived EVs. These observations suggest that the cellular characteristics of Huh7 and HepG2 cells may favour EV association, although the mechanisms responsible were not directly investigated. Previous research has implicated cellular endocytic activity and interactions with extracellular particles in EV uptake [30]. Such processes could contribute to the differences observed here, but the present experiments cannot establish whether the detected fluorescence resulted from surface binding, internalization, or a combination of both. Although previous in vivo studies have reported substantial EV accumulation in the liver [31], organ-level accumulation should not be interpreted as direct evidence of EV internalization by hepatocytes or liver-derived tumour cells.

In contrast, parental HEK293T cells consistently exhibited relatively low percentages of fluorescence-positive cells following exposure to their own EVs. This observation suggests that sharing a cellular origin does not necessarily result in high EV association under the experimental conditions examined. Several factors could potentially contribute to these differences, including the availability of cell-surface binding partners, membrane composition, and endocytic activity [30]. However, these possibilities remain speculative because receptor expression, membrane properties, and intracellular trafficking were not directly assessed.

Among the immune cell lines, THP-1 cells showed particularly high percentages of EV-associated fluorescence, exceeding those observed in Jurkat and HEK293T cells. At the highest EV dose, 80.3% and 84.7% of THP-1 cells were fluorescence-positive after 2 h and 4 h, respectively. Jurkat cells also exhibited substantial fluorescence, although the percentages changed relatively little between the two incubation periods at higher EV doses. Monocyte-lineage cells are known to possess cellular machinery involved in particle recognition and endocytosis [32,33]. These established biological properties offer a possible explanation for the relatively high fluorescence observed in THP-1 cells, but the present results do not demonstrate that phagocytosis or another specific internalization pathway was responsible. From a translational perspective, the differences observed between immune cell types highlight the importance of examining EV interactions with immune populations when evaluating candidate delivery systems. Nevertheless, whether these interactions affect functional cargo delivery remains unknown.

The murine melanoma experiments further demonstrated differences in EV-associated fluorescence between recipient-cell lines. Following exposure to HEK293T-derived EVs, Yummer cells exhibited higher percentages of fluorescence-positive cells than B16F10 cells at both examined time points. This finding indicates that substantial variation can occur even between cell lines derived from the same broad tumour category.

Comparisons across EV donor-cell sources revealed similar patterns of recipient-cell association, with Huh7 and HepG2 cells exhibiting relatively high EV-associated fluorescence following exposure to HEK293T-, SKBR-3-, and Yummer-derived EVs. These observations highlight the potential importance of recipient-cell identity in EV association. However, the unbalanced donor–recipient combinations prevent determination of the relative contributions of donor-cell origin and recipient-cell characteristics. Furthermore, the relatively high EV-associated fluorescence observed in Huh7 and HepG2 cells warrants further investigation to establish whether these interactions result in EV internalization and functional cargo delivery.

Interestingly, whereas HEK293T and SKBR-3 cells exhibited relatively low association with their respective EVs, Yummer cells showed significantly higher EV-associated fluorescence following exposure to Yummer-derived EVs than B16F10 cells. This indicates that low parental-cell association is not universal and suggests that donor–recipient interactions may influence EV association. Differences in membrane properties or cell-surface recognition molecules could contribute to these observations, although further mechanistic studies are required.

Dose- and time-dependent increases in EV-associated fluorescence were consistently observed across several recipient-cell lines. These findings are consistent with concentration- and exposure-dependent EV–cell interactions. However, such patterns cannot, by themselves, establish active internalization, because increased surface association could produce similar changes in fluorescence. In some cell lines, the percentages of fluorescence-positive cells changed only slightly between higher EV doses. This apparent plateau may reflect a high proportion of cells already exceeding the fluorescence-detection threshold, rather than saturation of EV binding sites or intracellular processing capacity. Further experiments measuring fluorescence intensity, surface-bound versus internalized EVs, and functional cargo transfer would be required to distinguish these possibilities. Accordingly, the present data cannot determine whether increasing EV doses beyond the tested range would improve functional delivery efficiency [34]. This distinction is particularly relevant to the therapeutic application of EVs, for which efficient cellular association does not necessarily translate into successful intracellular cargo delivery or therapeutic efficacy [35].

Several limitations should be acknowledged. First, EV association was assessed by flow cytometry following incubation with mNG-CD81-labelled EVs and washing. This approach cannot distinguish surface-bound from internalized EVs; therefore, EV-associated fluorescence should not be interpreted as a direct measure of internalization efficiency. Second, the mechanisms underlying recipient-cell-specific EV association were not directly investigated. Differences in membrane composition, surface charge, glycocalyx, membrane fluidity, receptor expression, endocytic pathways, and intracellular trafficking may contribute to the observed patterns [36,37,38]. Cell size may also influence EV association, although supporting evidence from nanoparticle studies is indirect [39]. Third, the donor–recipient combinations were not fully balanced, preventing determination of the relative contributions of donor-cell origin and recipient-cell characteristics. Fourth, stable expression of mNG-CD81 may affect EV biogenesis, membrane composition, and interactions with recipient cells. As labelled EVs were not directly compared with unmodified EVs, their structural and functional equivalence cannot be assumed. Furthermore, the experiments were conducted exclusively in vitro and previously reported hepatic EV accumulation and immune-cell interactions in vivo [40,41] do not necessarily reflect the cellular patterns observed here. Finally, functional cargo delivery and therapeutic efficacy were not evaluated. Future studies should incorporate direct internalization assays, mechanistic investigations, balanced donor–recipient comparisons, and in vivo validation. Comparisons between EVs from parental and mNG-CD81-expressing donor cells are also needed to assess potential effects of fluorescent tagging on EV yield, size distribution, marker abundance, and recipient-cell association.

Collectively, these findings demonstrate that EV-associated fluorescence varies substantially across recipient-cell populations and that similar recipient-cell patterns can occur with EVs from different donor sources. They also highlight the need to distinguish cell-surface association from internalization and functional delivery when evaluating EV-based delivery platforms. Future work integrating balanced donor–recipient comparisons, mechanistic studies, functional cargo-delivery assays, and in vivo validation will be essential to determine how these observations translate into effective and selective EV-based therapeutic strategies.

## 5. Conclusions

This study highlights recipient-cell specificity as a key feature of EV–cell interactions, demonstrating substantial differences in EV-associated fluorescence across human epithelial, immune, and murine melanoma cell lines. Huh7, HepG2, and THP-1 cells generally exhibited high EV-associated fluorescence, whereas parental HEK293T and SKBR-3 cells showed relatively low association with their respective EVs. In contrast, Yummer cells exhibited greater association with Yummer-derived EVs than B16F10 cells, indicating that low parental-cell association is not universal. The recurrence of similar recipient-cell patterns across different EV donor sources further supports the importance of recipient-cell identity in shaping EV association, although the relative contributions of donor- and recipient-cell characteristics remain unresolved. EV-associated fluorescence generally increased with dose and incubation time. Importantly, these measurements do not distinguish surface association from internalization or establish functional cargo delivery. Together, these findings underscore the importance of considering recipient-cell specificity when designing EV-based delivery strategies. Further studies investigating the underlying mechanisms, EV internalization, functional cargo delivery, and in vivo behaviour are needed to establish their therapeutic relevance.

## Data Availability

The data that support the findings of this study are available from the corresponding author upon reasonable request.

## Supplementary Materials

The following supporting information can be downloaded at: https://www.mdpi.com/article/10.3390/cells15212010/s1.
