# Supplementary material for: Recipient Cell Type-Dependent Association of Extracellular Vesicles from Different Cellular Origins
Source: Cells. 2026 Nov 4;15(21):2010. doi: 10.3390/cells15212010 (PMC13762198; doi:10.3390/cells15212010)
Supplement: Supplementary file 1 [file cells-15-02010-s001.zip › cells-4597675-supplementary.pdf]

Supplementary Figures

Article

Title: Cell Type–Dependent Association of Extracellular Vesicles from Different Cellular Origins

Doste R. Mamand [1,2,3,]

[1]Department of Laboratory Medicine, Division of Biomolecular and Cellular Medicine, Karolinska Institute, Stockholm, Sweden.
[2]Biology Research center, Research Center, University of Zakho, Zakho, 42002, Duhok, Kurdistan Region, Iraq.
[3]Breast Center, Karolinska Comprehensive Cancer Center, Karolinska University Hospital, Stockholm, Sweden.

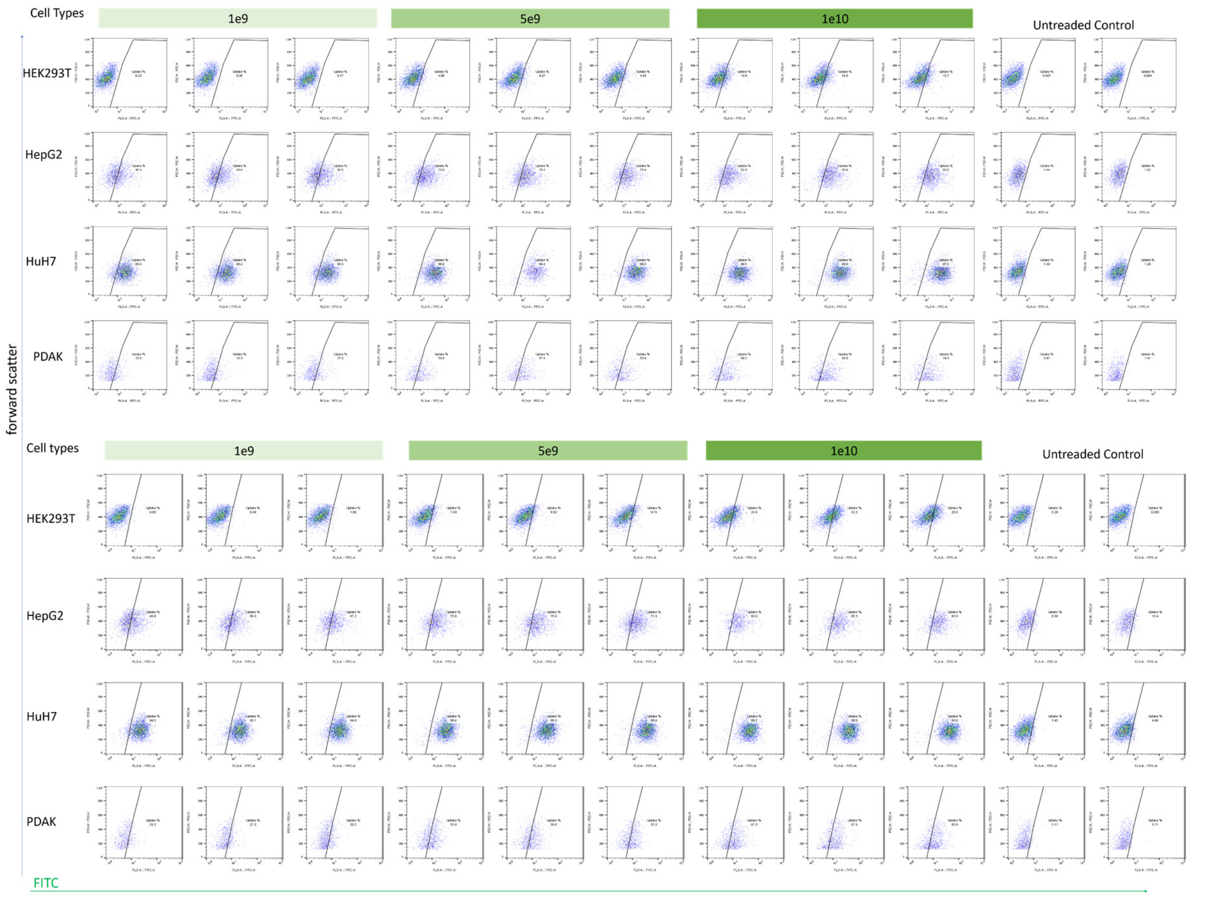

Supplementary Figure S1. Association of HEK293T-derived EVs with human epithelial cell lines. (Upper panel) Representative flow cytometry plots showing EV-associated fluorescence in HEK293T, PDAK, HepG2, and Huh7 cells following incubation with mNeonGreen (mNG)-CD81-labelled HEK293T-derived EVs at concentrations of 1×10^9, 5×10^9, or 1×10^{10} particles for 2 h. **(Lower panel)** Representative flow cytometry plots showing EV-associated fluorescence in the same recipient-cell lines following 4 h of incubation at the indicated EV concentrations. Untreated controls represent cells not exposed to EVs. Data are presented as mean ± standard deviation (SD) from three independent experiments (n = 3).

Supplementary Figures

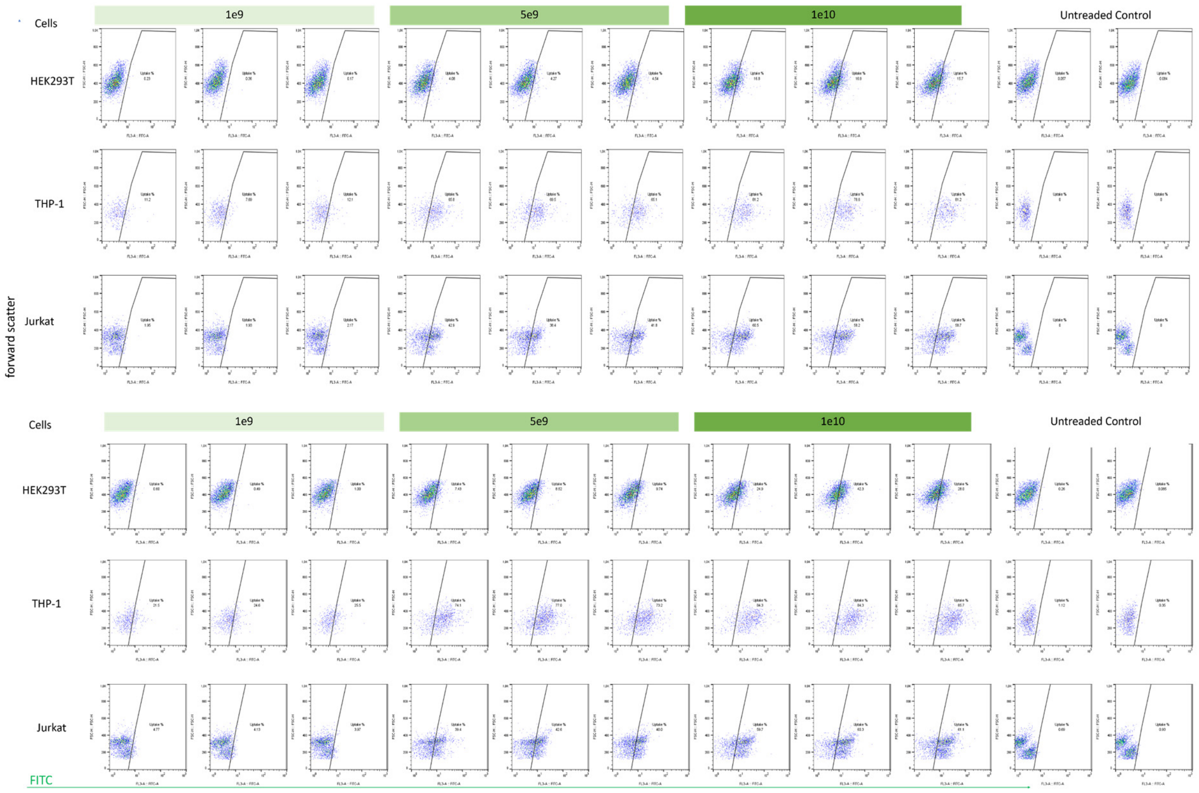

Supplementary Figure S2. Association of HEK293T-derived EVs with human immune cell lines. (Upper panel) Representative flow cytometry plots showing EV-associated fluorescence in Jurkat, THP-1, and HEK293T cells following incubation with mNG-CD81-labelled HEK293T-derived EVs at concentrations of 1×10^9, 5×10^9, or 1×10^{10} particles for 2 h. **(Lower panel)** Representative flow cytometry plots showing EV-associated fluorescence in the same cell lines following 4 h of incubation at the indicated EV concentrations. Untreated controls represent cells not exposed to EVs. Quantitative data are presented as mean ± standard deviation (SD) from three independent experiments (n = 3).

Supplementary Figures

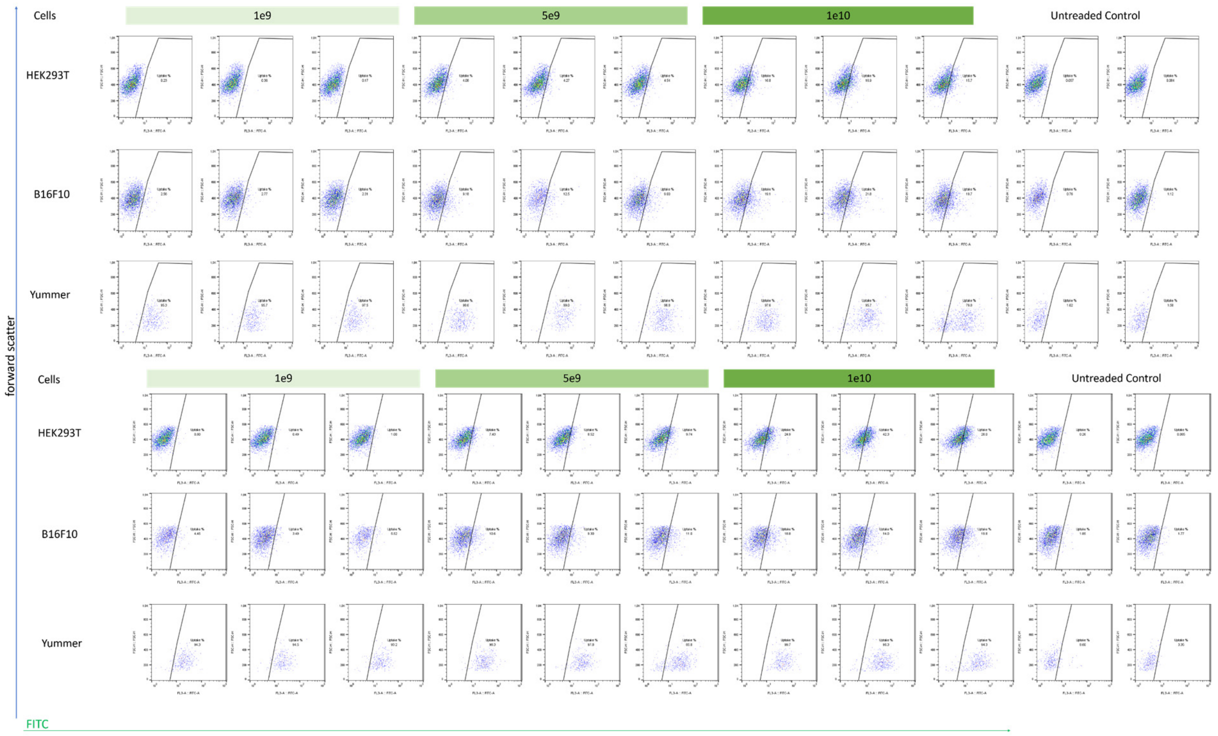

Supplementary Figure S3. Association of HEK293T-derived EVs with murine melanoma cell lines. (Upper panel) Representative flow cytometry plots showing EV-associated fluorescence in B16F10 and Yummer melanoma cells following incubation with mNG-CD81-labelled HEK293T-derived EVs at concentrations of 1×10^9, 5×10^9, or 1×10^{10} particles for 2 h. **(Lower panel)** Representative flow cytometry plots showing EV-associated fluorescence in the same cell lines following 4 h of incubation at the indicated EV concentrations. Untreated controls represent cells not exposed to EVs. Quantitative data are presented as mean ± standard deviation (SD) from three independent experiments (n = 3).

Supplementary Figures

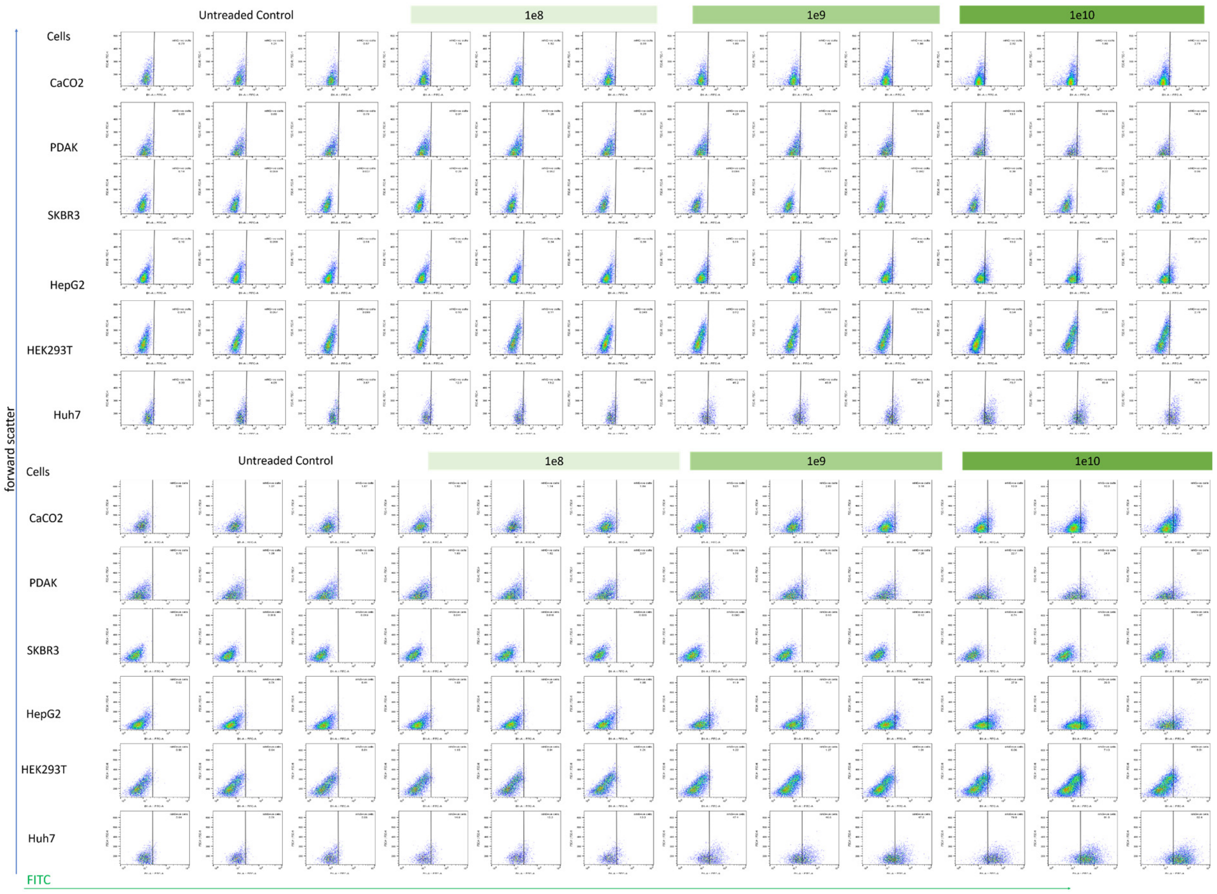

Supplementary Figure S4. Association of SKBR-3-derived EVs with human epithelial cell lines. (Upper panel) Representative flow cytometry plots showing EV-associated fluorescence in SKBR-3, HEK293T, Caco-2, PDAK, HepG2, and Huh7 cells following incubation with mNG-CD81-labelled SKBR-3-derived EVs at concentrations of 1×10^8, 1×10^9, or 1×10^{10} particles for 2 h. **(Lower panel)** Representative flow cytometry plots showing EV-associated fluorescence in the same recipient-cell lines following 4 h of incubation at the indicated EV concentrations. Untreated controls represent cells not exposed to EVs. Quantitative data, where shown, are presented as mean ± standard deviation (SD) from three independent experiments (n = 3).

Supplementary Figures

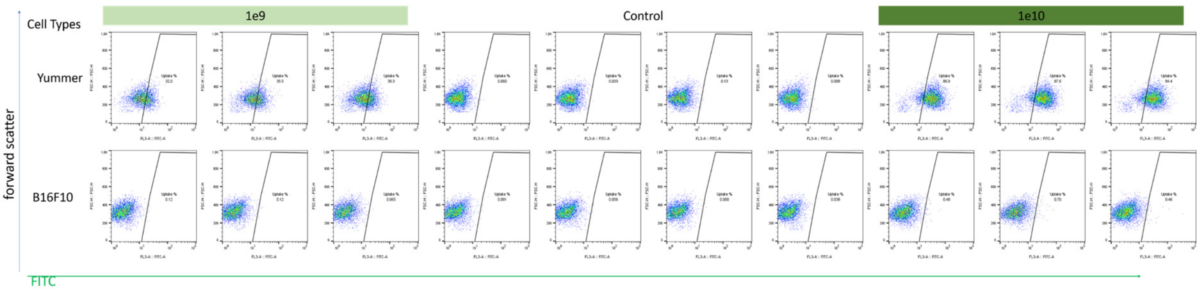

Supplementary Figure S5. Association of Yummer-derived EVs with murine melanoma cell lines. (Upper Pannel) Representative flow cytometry plots showing EV-associated fluorescence in B16F10 and Yummer cells following incubation with mNG-CD81-labelled Yummer-derived EVs at concentrations of 1×10^9 or 1×10^{10} particles for 2 h. **(Lowe Pannel)** Representative flow cytometry plots showing EV-associated fluorescence following 4 h of incubation at the indicated concentrations. Untreated controls represent cells not exposed to EVs. Quantitative data are presented as mean ± standard deviation (SD) from three independent experiments (n = 3).

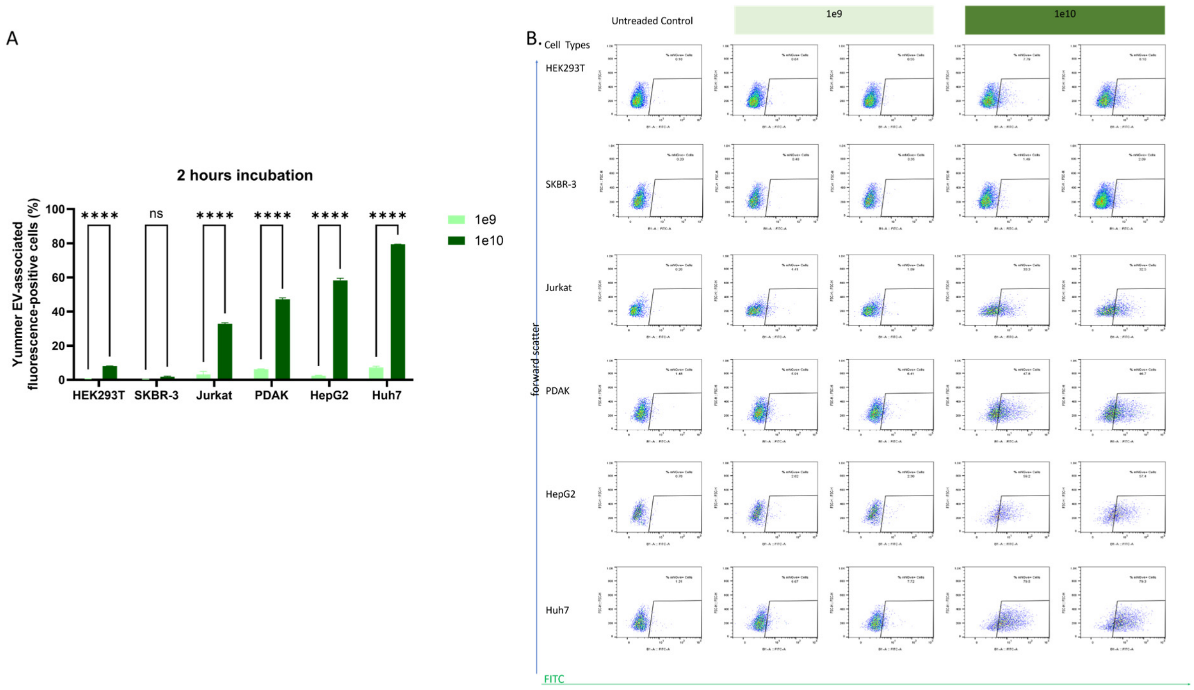

Supplementary Figure S6. Association of Yummer-derived EVs with human recipient-cell lines. Human HEK293T, SKBR-3, Jurkat, PDAK, HepG2, and Huh7 cells were incubated with mNG-CD81-labelled EVs derived from Yummer cells at different EV concentrations and incubation times. **(A)** Percentage of EV-associated fluorescence-positive recipient cells measured by flow cytometry. **(B)** Representative flow cytometry dot plots illustrating EV-associated fluorescence across the recipient-cell lines and experimental conditions. Quantitative data are presented as mean ± standard deviation (SD) from two independent experiments (n = 2).
